# Macrophages from Susceptible and Resistant Chicken Lines have Different Transcriptomes following Marek’s Disease Virus Infection

**DOI:** 10.3390/genes10020074

**Published:** 2019-01-22

**Authors:** Pankaj Chakraborty, Richard Kuo, Lonneke Vervelde, Bernadette M. Dutia, Pete Kaiser, Jacqueline Smith

**Affiliations:** 1 The Roslin Institute and R(D)SVS, University of Edinburgh, Easter Bush, Midlothian EH25 9RG, UK; pcb23m@yahoo.com (P.C.); richard.kuo@roslin.ed.ac.uk (R.K.); lonneke.vervelde@roslin.ed.ac.uk (L.V.); bernadette.dutia@roslin.ed.ac.uk (B.M.D.); 2 Chittagong Veterinary and Animal Sciences University, Khulshi, Chittagong 4225, Bangladesh

**Keywords:** chickens, Marek’s disease virus, disease resistance, macrophages, RNA-seq

## Abstract

Despite successful control by vaccination, Marek’s disease (MD) has continued evolving to greater virulence over recent years. To control MD, selection and breeding of MD-resistant chickens might be a suitable option. MHC-congenic inbred chicken lines, 6_1_ and 7_2_, are highly resistant and susceptible to MD, respectively, but the cellular and genetic basis for these phenotypes is unknown. Marek’s disease virus (MDV) infects macrophages, B-cells, and activated T-cells in vivo. This study investigates the cellular basis of resistance to MD in vitro with the hypothesis that resistance is determined by cells active during the innate immune response. Chicken bone marrow-derived macrophages from lines 6_1_ and 7_2_ were infected with MDV in vitro. Flow cytometry showed that a higher percentage of macrophages were infected in line 7_2_ than in line 6_1_. A transcriptomic study followed by in silico functional analysis of differentially expressed genes was then carried out between the two lines pre- and post-infection. Analysis supports the hypothesis that macrophages from susceptible and resistant chicken lines display a marked difference in their transcriptome following MDV infection. Resistance to infection, differential activation of biological pathways, and suppression of oncogenic potential are among host defense strategies identified in macrophages from resistant chickens.

## 1. Introduction

Marek’s disease (MD) is an oncogenic viral disease of chickens caused by the *Gallid alphaherpesvirus 2*, which is traditionally known as Marek’s disease virus (MDV). *Gallid herpesvirus 2* is part of the Alphaherpesvirinae subfamily in the genus *Mardivirus*. Losses incurred by the poultry industry due to the virus are considerable. Until now, effective vaccination has led to successful control of this disease, but as none of these vaccines can induce sterile immunity against MD, the virulence of MDV has been increasing over the years amid the introduction of new generations of vaccines [1]. This emphasizes the necessity for implementation of alternative control methods for MD. A suitable method could be the selection and breeding of MD-resistant chickens. The estimates of heritability of MD resistance are as high as 61% [2], suggesting good potential for genetic improvement for meat and egg production [3]. However, resistance to MD is multifaceted, as many features including host genetics, viral strain, and environmental factors constitutively regulate this mechanism. 

In the host, various genes from within and without the MHC (Major Histocompatibility Complex) region are involved. Within the MHC, the C-type lectin-like receptor genes, *B-NK* and *B-lec* have been considered as potential candidate genes for resistance to MD [4] and previous reports show that the C-type lectin-like receptors (*Ly49H*, *NKR-P1,* and *Clr*) in mouse and rat are associated with resistance to another herpesvirus, cytomegalovirus (CMV) [5,6]. Non-MHC genes also play a pivotal role in MD resistance. More than 20 separate QTL (quantitative trait loci) are involved [7,8,9]. This can be most clearly explained by studies from two inbred chicken lines (6_1_ and 7_2_), which are homozygous for the B2 haplotype [10]. Lines 6_1_ and 7_2_ are highly resistant and highly susceptible to clinical MD respectively, which is reflected by large differences in host viraemia levels when challenged with MDV [11,12], suggesting that resistance to MD is mostly exerted by the genes outside of the MHC. Several potential non-MHC candidate genes for MD-resistance or susceptibility have been identified in various cell types to date. These include *TLR3* and *IL6* in chick embryo fibroblasts [13], *GH1* and *Ly6E* in splenocytes [14,15] and *IRG1* in splenocytes [16]. Marek’s disease virus infects various lymphoid cells such as macrophages, B and T cells [17,18], as well as non-lymphoid cells such as fibroblasts [13], EACs (ellipsoid-associated cells) [19] in both in vivo and in vitro conditions, but it is still unclear at what stage of infection and in/or by which cells those resistance mechanisms or genes are expressed.

Resistance to MD in the inbred lines 6_1_ and 7_2_, in terms of viral load, is established as early as 3 dpi [16,20] and by this time the virus has infected phagocytic cells, B cells, and activated T cells [17]. Hence, the resistance mechanisms could be exerted in any or all of these cell types. The early events of differential viraemia [20] and gene expression profiles [16] in these two inbred lines suggest that the differences between the two lines are due to innate rather than adaptive host immune responses [21]. However, very little is known about the early stages of MDV infection. In order to explore the resistance phenotype in innate immune cells, bone marrow-derived macrophages (BMDMs) from lines 6_1_ and 7_2_ were infected with MDV using a newly developed in vitro MDV-phagocyte infection model [18]. Variation in gene expression is a major determinant of phenotypic variation and differences in MD genetic resistance are most likely due to variation in the transcriptional regulation of genes [22]. Therefore, RNA-seq and transcriptomic analysis were used to determine differentially expressed (DE) genes in the two chicken lines pre- and post-MDV infection of BMDMs. The aims of this study were to determine the resistance phenotype in macrophages from these two inbred chicken lines as well as to explore the biological pathways associated with resistance or susceptibility to MD.

## 2. Materials and Methods

### 2.1. Experimental Animals

Inbred specific pathogen free (SPF) chickens from lines 6_1_ and 7_2_ were used in this study. Line 6_1_ birds were bred in the Poultry Production Unit at the Institute for Animal Health, Compton, UK and reared in the poultry unit of The Moredun Research Institute, while line 7_2_ chickens were bred and reared at the National Avian Research Facility (NARF), Edinburgh, UK. For chicken embryos, chicken layer line J was used (an intercross bred from 9 lines, originally bred from Brown Leghorn chickens at the Poultry Research Centre, Edinburgh). They were bred and conventionally raised at the NARF (http://www.narf.ac.uk/chickens/lines).

### 2.2. Marek’s Disease Virus

The virus, CVI988 *UL41* eGFP, was generated from a BAC construct of vaccine strain CVI988 (Rispens) of MDV serotype 1, in which the *UL41* gene was replaced with eGFP (enhanced Green Fluorescent Protein) under control of the murine phosphoglycerol kinase promoter [23]. *UL41* is a non-essential gene for MDV replication and a *UL41*-deletant mutant replicates as well as the parental strain in vitro [24]. The presence of eGFP will therefore indicate MDV replication.

### 2.3. Cell Cultures

Chicken embryo fibroblasts (CEFs) were cultured from 9–11-day-old chicken embryos in T_175_ flasks at 38.5 °C with 5% CO_2_ in CEF medium consisting of M-199 medium (Gibco) containing 10% (*v*/*v*) tryptose phosphate broth (Invitrogen), 2.7% (*v*/*v*) NaHCO3 (Sigma–Aldrich), 1% (*v*/*v*) pen-strep (Sigma–Aldrich), 0.5% (*v*/*v*) gentamycin (Sigma–Aldrich), 0.001% (*v*/*v*) fungizone (amphotericin B, 250 μg/mL) (Thermo Scientific), and 0.5–10% (*v*/*v*) fetal bovine serum (FBS) (Gibco) depending on CEF confluency in culture flasks. The MDV-BAC virus was initially grown and propagated in CEF cultures as previously described [25]. The MDV-infected CEFs were then grown in large numbers and pooled together to obtain a high virus titer. Pooled infected CEFs were resuspended in freezing media (FBS (PAA), RPMI-1640 (Sigma–Aldrich) and DMSO), aliquoted (250–500 μL/cryovial) and stored at −80 °C until further use.

Chicken bone marrow cells were isolated from 3 to 6-week-old birds and BMDMs were cultured as described previously [26]. In order to obtain approximately 1 × 10^7^ BMDMs at harvest, 4 day culture bone marrow (BM) cells were seeded at a concentration of approximately 1 × 10^6^ cells/mL. Cells were cultured in T_75_ flasks at 41 °C with 5% CO_2_ using RPMI-1640 medium supplemented with 10% heat-inactivated FBS, 1% l-glutamine and 0.1% pen-strep. Recombinant chicken colony stimulating factor 1 (chCSF-1) [26] was added to the BMDM cultures and medium was refreshed every 2 days.

### 2.4. Co-Culture Infection Experiments and Fluorescence Activated Cell Sorting (FACS)

Due to the cell-associated nature of MDV, infected CEFs were used to infect macrophages. On the day of macrophage infection, infected CEFs were harvested by treatment with 2.5% trypsin (diluted in PBS), pelleted by centrifugation (500× *g* for 5 min) and resuspended in FACS buffer (PBS and 1% BSA). In order to remove macrophages from the CEF culture, they were stained with anti-CD45 (clone AV53, isotype IgG1, The Pirbright Institute) and a goat anti-mouse IgG1 conjugated with Alexa Fluor (AF) 647 as secondary antibody and Gr 13.1 as isotype control (ovine NKp46; kindly provided by Dr. Timothy Connelly, The Roslin Institute) according to the procedures described previously [27]. The GFP^+^ CD45^-^ CEFs were sorted using the FACSAriaTM III cell sorter (BD Biosciences). Data were analyzed using FACSDiva v 6.1.3 software.

The BMDMs were infected with 2 × 10^6^ sorted infected CEF on day 4 of culture in T_75_ flasks at an infection ratio of 1:5 (CEF:BMDM) in RPMI-1640 medium containing 10% FBS (Gibco), 1% pen-strep, and 1% l-glutamine. Co-cultured cells were incubated at 41 °C with 5% CO_2_ for 1 day and harvested for downstream experiments.

### 2.5. Flow Cytometry

Cells were harvested with 100 mM EDTA in PBS, pelleted by centrifugation and resuspended in PBS containing 1% BSA and 0.1% sodium azide. Immunofluorescent staining was carried out using a monocyte/macrophage marker (clone KUL01, isotype IgG1, Southern Biotech) and anti-CD45. KUL01 was recently identified as a mannose receptor MCRL1B [28]. Cells were stained for flow cytometric analysis as described above and analyzed using a FACSCalibur (BD Biosciences). Viable cells were gated based on 7-AAD (7-aminoactinomycin D, Life Technologies) staining and the resulting data were analyzed with FlowJo software.

### 2.6. Real-Time Quantitative RT-PCR

Total RNAs were extracted using RNeasy Mini Kits (Qiagen). Cytokine mRNA expression levels were assessed using TaqMan real-time quantitative RT-PCR (qRT-PCR) by a well-described method [29,30] using *28S* RNA as the reference gene [31]. Primers and probes used in this study for cytokines and *28S* RNA-specific amplification are given in Table 1. The results are expressed as 40-Ct by deducting each Ct value from the total number of cycles (40). All data were checked for normality and pairwise statistical comparisons between means in two groups (control v infected, control v control, infected v infected) of the two inbred chicken lines (6_1_ and 7_2_) were carried out with a 2-Sample *t*-test (95% confidence interval) using Minitab 18 software (State College, USA). Statistical significance was determined as *p* < 0.05.

### 2.7. Cells and Sample Preparation for RNA-sequencing (RNA-seq)

The BMDMs from inbred chicken lines 6_1_ and 7_2_ were infected with pre-sorted MDV-infected CEFs at an infection ratio of 1:5 (CEF:BMDM) as described previously [18]. Upon infection, control and infected BMDMs were sorted on 1 dpi based on eGFP and *CD45* expression. The RNAs were extracted using RNeasy Mini Kits (Qiagen, UK) and DNase treatment of RNAs was carried out with Ambion Turbo DNA-free kits (Life Technologies). The RNA quantification was performed using Qubit RNA Assay kit with the Qubit 2.0 Fluorometer (Invitrogen). Three separate biological replicates for each of the control and infected BMDMs were used for RNA preparation and subsequent sequencing.

### 2.8. RNA-seq and Analysis

RNA sequencing was performed by the Edinburgh Genomics sequencing facility (Edinburgh, UK) using a pool of individually barcoded RNA samples representing 3 biological replicates in each of the control and infected BMDMs. Paired-end sequencing was carried out on an Illumina HiSeq 2500 using an Illumina TruSeq Rapid SBS Kit (Illumina, Little Chesterford, UK). The quality of the RNA-seq reads was evaluated using the software FastQC [32]. Adapter sequences (Illumina TruSeq v3 adapters) were trimmed from the FASTQ sequences using Cutadapt software [33] with a minimum length cut-off at 50 bp. Trimmed reads were analysed to explore the differentially expressed (DE) genes in MDV-infected and control BMDMs. The data analysis pipeline included the following steps: The short reads were aligned to the chicken genome sequence (Galgal4, Ensembl release 78) using Bowtie and TopHat software packages [34]. Following alignment of the RNA-seq reads, HTSeq-count was used to calculate counts per million (CPM) for each gene [35]. The differential gene expression was then analysed using edgeR (empirical analysis of digital gene expression in R) [36,37]. Differentially expressed genes were filtered using an FDR (False discovery rate) of 0.05 and fold change > 2. Viral transcript expression was measured by mapping reads to the CVI988 MDV genome (Accession: DQ530348) and counting reads using Kallisto (https://pachterlab.github.io/kallisto/). Sleuth (https://pachterlab.github.io/sleuth/) was then used to measure differential expression between lines. Expression is detailed as transcripts per million (TPM) values.

### 2.9. Functional Analysis of DE Genes

Significant DEGs (*p* < 0.05) were submitted to the DAVID (Database for Annotation, Visualization and Integrated Discovery) software package (version 6.7) (http://david.abcc.ncifcrf.gov/) to identify enriched gene ontology terms associated with genes expressed during the host response in each line of birds. The analysis classification stringency was set to a high level and FDR for multiple testing was performed by the Benjamini and Hochberg method invoked within DAVID [38]. The enrichment score was calculated with a set of input genes highly associated with certain biological terms, which was statistically measured by a Fisher Exact test in the DAVID system (*p* < 0.05). The overall enrichment score for the group is based on the *p*-value of each term member.

To determine which biological pathways are associated with DEGs identified pre- and post-MDV infection in each line, the Pathway Express software within the Onto-Tools suite was used [39]. Genes differentially expressed in the control and MDV-infected macrophages (*p* < 0.05) were analyzed against a reference list of genes based on Galgal4 (Ensembl release 78). Annotation is based upon the equivalent human genes. In this program, the analysis displays up- and downregulated genes on KEGG (Kyoto Encyclopedia of Genes and Genomes) pathways. The impact factor analysis includes the classical statistics as well as other crucial factors such as the magnitude of each gene’s expression change and its type, position, and interaction in the given pathways. Significance is determined using the FDR-corrected gamma *p*-value (<0.05), which is determined based on the impact analysis of each gene’s expression and interactions in a given pathway. Gene networks involved in a particular experimental condition are established using pathway diagrams. iPathwayGuide v1.2 (https://www.advaitabio.com/ipathwayguide.html) was also used to show genes up- and downregulated in significant pathways. This software analysis tool implements the *“Impact Analysis”* approach that takes into consideration the direction and type of all signals on a pathway, the position, role, and type of every gene, etc., as described above. The Ingenuity Pathway Analysis (IPA) program (Ingenuity Systems, http://www.ingenuity.com/) was used to reveal which canonical pathways (see Supplementary Materials, Figure S1) and biological functions (see Supplementary Materials, Figure S2) are inherently active in the control BMDMs between the two lines and which are switched on following MDV infection in the host. The *p*-value was calculated using the right-tailed Fisher Exact Test (threshold *p* < 0.05).

Genes uniquely expressed in macrophages of the resistant (6_1_) and susceptible (7_2_) lines during the host response were analyzed for enriched biological pathways and transcription factor binding sites using the Expander (v7.11) software package (http://acgt.cs.tau.ac.il/expander/expander.html). The enrichment of transcription factor binding sites (TFBS) was carried out by using the PRIMA algorithm, included within the Expander package. Significance was determined as Bonferroni corrected *p*-values lower than 1 × 10^−4^.

### 2.10. Determining MDV Quantitative Trait Loci (QTL) Candidate Genes

The BioMart data mining tool within the Ensembl database (release 78) (http://www.ensembl.org/index.html) was used to identify genes located in genomic regions previously identified (see Supplementary Materials, Table S1) as being under QTLs for MDV resistance [7,8,9].

## 3. Results

### 3.1. Infection of Macrophages from Susceptible and Resistant Birds

**Figure 1:**
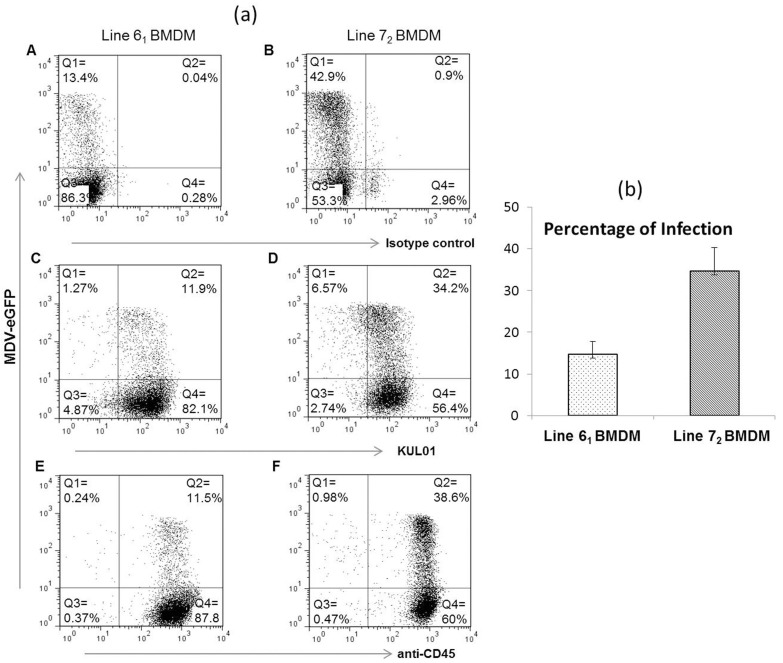
(**a**) The in vitro infection of macrophages from two inbred lines with Marek’s disease virus (MDV). Chicken bone marrow-derived macrophages (BMDMs) from lines 6_1_ and 7_2_ were cultured with Colony stimulating factor 1 (CSF-1) for 4 days. On the day of infection, pre-sorted eGFP^+^ CD45^-^ chick embryo fibroblasts (CEF)s were co-cultured with BMDMs at a ratio of 1:5 (CEF:BMDM). After 1 day in culture, flow cytometric characterization of in vitro-infected BMDMs was performed for the surface expression of KUL0*1* and *CD45*. Data shown are representative of three independent experiments. Distribution of cells: Q1, infected CEFs; Q2, infected BMDMs; Q3, uninfected CEFs; Q4, uninfected BMDMs. **Panel A** and **B**: staining with isotype control for line 6_1_ and 7_2_; **Panel C** and **D**: staining with KUL01 for line 6_1_ and 7_2_; **Panel E** and **F**: staining with CD45 for line 6_1_ and 7_2_. (**b**) Graph showing means and the respective standard error of mean (SEM) of the percentages of infected cells found within the three biological replicates from lines 6_1_ and 7_2_ BMDM during cell sorting experiments.

Chicken BMDMs from lines 6_1_ and 7_2_ were co-cultured with pre-sorted MDV-infected CD45^-^ GFP^+^ CEFs at the same ratios (1:5) as described previously [18]. Flow cytometric characterization of BMDMs with KUL01 and CD45 staining at 1 dpi revealed that the proportion of infected macrophages was around three times higher in line 7_2_ (34–38%), which is susceptible to MDV, than in line 6_1_ (11–12%), which is resistant to MDV infection (Figure 1a). Co-culture cell sorting experiments also revealed similar differences between the infected BMDMs of the two inbred lines (Figure 1b). 

### 3.2. Pro-Inflammatory Cytokine Expression

**Figure 2:**
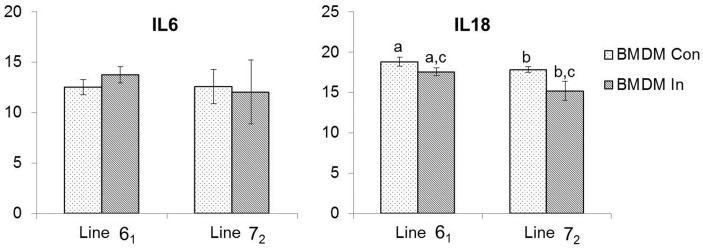
Quantification of pro-inflammatory cytokines by qRT-PCR. The mRNA expression levels of *IL6* and *IL18* between control and flow sort purified infected BMDMs of lines 6_1_ and 7_2_ were measured on 1 dpi. Pairwise statistical comparisons between means in two groups (*n* = 3 in each group) of the two inbred chicken lines were carried out with a two-sample *t*-test (95% confidence interval) using Minitab 18 software. Con = control; In = infected. Means that share the same letter are significantly different (*p* < 0.05).

The mRNA expression levels of two pro-inflammatory cytokines, *IL6* and *IL18*, were measured at 1 dpi between flow sort purified MDV-infected and control BMDM from the two inbred lines using TaqMan qRT-PCR. In general, the expression levels of the two cytokines were lower in line 7_2_ BMDM following MDV infection compared to line 6_1_ (Figure 2). There was no statistically significant difference in the mean *IL6* mRNA expression in any of the BMDM groups from the two inbred lines. The overall expression of *IL18* mRNA was significantly lower in infected BMDM than that of controls in each line. Although there was no significant difference in inherent *IL18* levels between the two lines, the expression of *IL18* was seen to be decreased significantly (*p* < 0.05) in the susceptible line (7_2_) compared to the resistant line (6_1_) following MDV infection (Figure 2).

### 3.3. Analysis of Gene Expression in Susceptible and Resistant Lines

Analysis of RNA-seq data was carried out to explore differences in gene expression in control and infected BMDMs from two inbred lines. The infected BMDMs were purified using flow sorting based on expression of eGFP-MDV and CD45. Comparisons were made as follows: (1) control BMDM (line 6_1_) v control BMDM (line 7_2_) to see which genes are inherently differentially expressed between susceptible and resistant birds; (2) control BMDM v infected BMDM to define the host response to infection in each line of birds; (3) (infected BMDM—control BMDM) from line 6_1_ v (infected BMDM—control BMDM) from line 7_2_ to examine differences in host response to MDV infection between susceptible and resistant birds. The numbers of genes with a fold change >2 (FDR < 0.05) used for downstream functional analyses are provided in Table 2. The full lists of DEGs in BMDMs from lines 6_1_ and 7_2_ are provided in Supplementary Materials, Table S2.

### 3.4. Inherent Differences in Gene Expression between the Two Lines

Differences in gene expression were observed between the two lines even before infection (360 DE genes in line 6_1_ and 729 DE genes in line 7_2_) (see Supplementary Materials, Table S2). Several genes that are known to be involved in the innate immune response were more highly expressed in the resistant line (6_1_) than in the susceptible line (7_2_). These included interferon regulatory factors (*IRF1, IRF6*); tumor necrosis factor alpha-induced proteins (*TNFAIP2, TNFAIP6*), TNF receptor associated factor and its interacting protein (*TRAF2*, *TRAF3IP2*); chemokine and chemokine receptor (*CXCL14, CCR2*); interferon induced transmembrane protein (*IFITM3*); and the myxovirus resistance gene (*MX1*). Conversely, DE genes with known immune function highly expressed in line 7_2_ included a member of the TNF superfamily (*TNFSF15*), interleukin and interleukin receptors (*IL1β, IL17RE, IL17RC*). Thus, we show the inherent differences in the expression of immune genes existing between the two lines.

### 3.5. Host Response to MDV Infection in Resistant and Susceptible Lines

A total of 479 genes were upregulated and 786 genes were downregulated in the MD-resistant line (6_1_) following MDV infection (see Supplementary Materials, Table S2). Few genes with known immune function were upregulated. Those that were, included a Tumour necrosis factor (TNF) superfamily member (*TNFSF13B*) and the suppressor of cytokine signaling gene (*SOCS7*). On the other hand, a number of immune related genes were downregulated in this line, such as TNF proteins and a TNF super family member (*TNFAIP3, TNFAIP6, TNFRSF6B*); Toll-like receptor (*TLR15*); chemokine and chemokine receptors (*CXCL13L2, CCR5, CXCR4*); interleukins and interleukin receptors (*IL4I1, IL12B, IL13RA2, IL21R, IL22RA2*); immunoglobulin superfamily member (*IGSF1*); janus–kinase gene (*JAK3*) and the lysozyme gene (*LYZ*).

In the MD-susceptible line (7_2_), 1133 genes were upregulated, and 1394 genes were downregulated after infection (see Supplementary Materials, Table S2). Genes with high expression levels included several chemokines and chemokine receptor (*CCL1, CCL4, CCL20, CX3CL1, CCR7*); interleukin and interleukin receptors (*IL2RA, IL8L1, IL20RA, IL23R*); TNFs (*TNFAIP2, TNFRSF4, TRAF1, TRAF3, TRAF5, TRAF3IP2*); genes involved in controlling the interferon response (*IFITM3, IFITM5, IRF1, IRF4, IRF6, IRF10, STAT1, STAT2, STAT4, BATF*); inducible nitric oxide synthase (*INOS*); *IGSF3* and nuclear factor kappa B subunit 2 gene (*NFKB2*). Downregulated genes in this line included some interleukins and interleukin receptor (*IL8, IL15, IL2RG*); interferon receptors and interferon controlling genes (*IFNAR1, IFNAR2, SOCS2, SOCS3*, *SOCS5*); *TNFSF15* and the avidin gene (*AVD*).

### 3.6. Differences in Host Response between the Two Lines Following MDV Infection

Differences in gene expression levels were identified between MDV-infected BMDMs from the two lines (401 DEGs were highly expressed in line 6_1_ compared to line 7_2_ and 555 DE genes were expressed at a higher level in line 7_2_ than in line 6_1_) (see Supplementary Materials, Table S2). Only a few immune genes were highly expressed in the resistant line (6_1_) compared to the susceptible line (7_2_). These included *TNFSF11, CXXC5, IL15, MX1,* and *LY6E*. In contrast, a number of chemokines and chemokine receptor (*CCL20, CX3CL1, CXCL13L2, CCR7*); interleukin and interleukin receptors (*IL8L1, IL1R2, IL2RA, IL17RE, IL20RA, IL23R*); and some other immune related genes (*STAT2, STAT4, IGSF3, BATF3*, *AMIGO2, LYZ*) were more highly expressed in the susceptible line (7_2_). Therefore, MDV infection is seen to drive differential upregulation of immune genes between resistant and susceptible lines.

### 3.7. Viral Gene Expression

Viral gene expression after infection in lines 6_1_ and 7_2_ was determined and is presented in Supplementary Materials, Table S3. The small number of reads seen in control samples are background. A high level of viral expression is seen in the infected samples. Differences in expression of the viral genes in each of the two lines is shown in Supplementary Materials, Table S4. Viral transcripts are found to be present at up to three times higher levels in line 7_2_ as compared to line 6_1._

### 3.8. Gene Ontology Analysis of Differentially Expressed (DE) Genes

Gene ontology analysis of DE genes was performed in order to understand the biological processes involved in BMDMs of resistant (6_1_) and susceptible (7_2_) birds following MDV infection. In line 6_1_, genes clustered into the following ontologies: carbohydrate binding, glycosylation, cell adhesion, and cell communication; whereas in line 7_2_ genes were associated with immune cell activation/differentiation, cytokine binding and signal transduction (see Supplementary Materials, Tables S5 and S6).

### 3.9. Functional Analysis Reveals Significantly Regulated Biological Pathways

**Figure 3:**
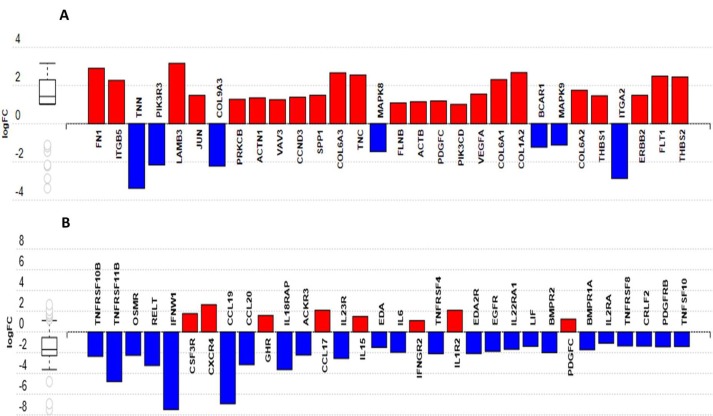
Differentially expressed genes in pathways responding to MDV infection as identified by iPathwayGuide. Up- and downregulated genes in (**A**) MD-resistant line 6_1_ and in (**B**) MD-susceptible line 7_2_ BMDMs are associated with focal adhesion and cytokine–cytokine receptor interaction pathways, respectively. Blue = upregulated; red = downregulated.

The DE genes were analyzed using iPathwayGuide to determine the biological pathway(s) altered during the host response following MDV infection in each line. In the resistant line (6_1_), focal adhesion was the most significantly altered biological pathway; whereas the cytokine–cytokine receptor interaction pathway was significantly perturbed in the susceptible line (7_2_). Figure 3a,b show the genes with altered expression patterns in the given biological pathway in BMDMs of lines 6_1_ and 7_2_, respectively.

**Figure 4:**
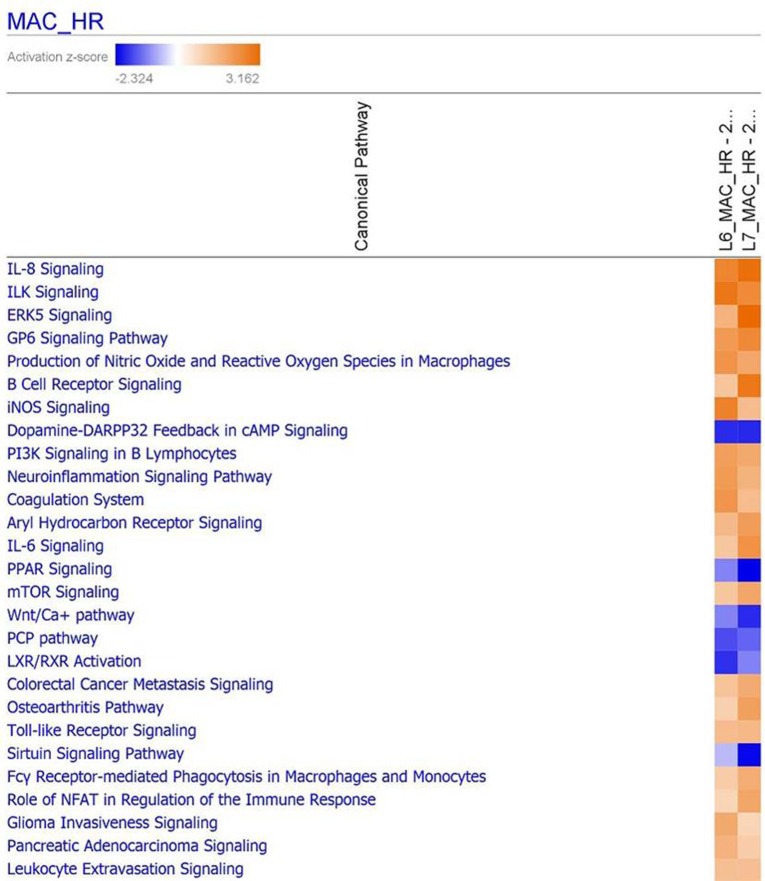
Functional analysis of DE genes using IPA (ingenuity pathway analysis). IPA showing the comparison of the most highly represented canonical pathways due to MDV infection in line 6_1_ and line 7_2_ BMDMs. MAC = macrophage; HR = host response.

Complementary to the findings from iPathwayGuide, use of the ingenuity pathway analysis (IPA) program revealed which canonical pathways were differentially regulated during MDV infection in BMDMs from each line (Figure 4). A higher response was seen in the resistant line (6_1_) for ILK signaling, iNOS signaling, coagulation, complement and TNFR signaling; while in the susceptible line (7_2_) a higher response was observed for IL8, ERK5, B-cell receptor (BCR) and IL6 signaling. Likewise, in line 6_1_ LXR/RXR activation and p53 signaling are lower than in line 7_2_, with PPAR signaling and sirtuin signaling lower in line 7_2_ than in line 6_1_.

**Figure 5:**
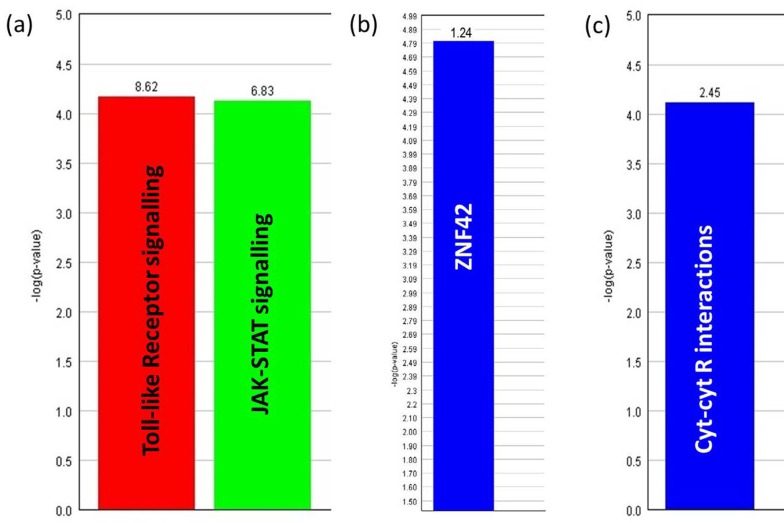
Over-representation analysis of genes with their involvement in biological processes as determined by Expander software. (**a**) The GO (gene ontology) processes involved with genes downregulated only in line 6_1_ infected BMDM and (**c**) the GO process involved with genes upregulated only in line 7_2_ infected BMDM. The frequency of genes of a functional class within the examined set is described as a percentage of the total. (**b**) Significantly enriched ZNF42 transcription factor (TF) binding sites in genes downregulated in line 6_1_ infected BMDM. The frequency ratio (frequency of the set divided by the frequency of the background) was 1.24, which means presence of 1.24 times as many ZNF42 sites than would be expected by chance.

Enriched gene ontology functional annotation was determined using the Expander program. Over-represented transcription factor binding sites (TSBFs) were also determined using this software. A set of genes downregulated only in line 6_1_ after MDV infection (see Supplementary Materials, Table S7) were seen to be involved with TLR signaling and JAK-STAT signaling pathways (Figure 5a). Both TLR and JAK-STAT signaling are important cellular pathways most likely to be involved in the innate immune response [16]. A substantial number of these genes had ZNF42 (zinc finger protein 42) transcription factor (TF) binding sites in their promoter regions (Figure 5b). The ZNF42 protein, also known as MZF1 (myloid zinc finger 1), is a putative oncogenic protein [40]. On the other hand, line 7_2_ had upregulated expression of a different set of genes (see Supplementary Materials, Table S8) which were seen to be associated with cytokine–cytokine receptor interactions (Figure 5c) which is also an important pathway involved in innate immunity [16]. No specific enrichment of TF binding sites was identified in this line.

### 3.10. Identification of Putative Candidate Genes for Resistance to MDV

By analyzing genes showing high levels of differential regulation after MDV infection (or differential inherent expression between lines) and mapping them to regions underlying known QTL, we were able to highlight potential candidate genes for resistance to MDV infection (Supplementary Materials, Table S9). These include the tumor suppressor gene, *RASEF* (expressed only during the line 6_1_ host response) and a gene involved in regulating cell-mediated immunity, *HHLA2* (highly expressed in line 7_2_ following infection). Inherent expression differences were also observed between the two lines such as that of a gene involved in formation of tight junctions (*CLDN5*). Several immune-related genes show different basal levels of gene expression. These include *CHGA* (also associated with neuroendocrine tumors), *CFH*, *SPP1,* and an immunoglobulin-like receptor. Genes with neuronal association (*SLC5A7, PAX6, NTN4L*) were seen to be upregulated in line 6_1,_ while a gene associated with lymphoma and leukemia (*CD72*) was highly expressed in line 7_2_.

## 4. Discussion

In order to explore the cellular basis of resistance to MD, BMDMs from two inbred chicken lines (6_1_ and 7_2_) were infected with MDV-infected CEF using a newly developed infection model and subsequently characterized by flow cytometry, qRT-PCR and RNA-seq on 1 dpi. Herpesviruses commonly infect and replicate in epithelial cells prior to infection of leukocytes. Chick embryo fibroblasts are comprised of a variety of cells types and hence infection with CEF-derived MDV likely represents the in vivo infection. The chicken inbred lines 6_1_ and 7_2_ are highly resistant and susceptible to MD, respectively, although they share the same MHC. Mortality rates by MD range from 7 to 94 per cent in resistant and susceptible chickens, respectively [21].

Pathologically the disease is manifested by T cell latency in resistant birds and by latency as well as lymphomatosis in susceptible birds. The two inbred lines (6_1_ and 7_2_) show differences in viraemia level (10-fold higher virus titre in susceptible compared to resistant birds) and gene expression profiles in splenocytes from the very early stages of MDV infection [11,16], suggesting that the inherent difference between the two lines is due to innate rather than the adaptive immune responses [21]. The cells of the innate immune system, especially macrophages, play a crucial role during MDV infection. For example, peritoneal macrophages isolated from MDV-infected chickens inhibit the formation of MDV plaques in vitro [41]. Peritoneal macrophages also show more phagocytic and plaque-inhibiting activity following MDV infection in susceptible birds compared to resistant chickens [42]. Macrophages from outbred chickens were shown to become infected in vitro in our previous study [18]. In the present study, BMDMs from MHC-congenic MD-resistant (6_1_) and susceptible (7_2_) inbred chickens were infected with MDV in vitro in the first study of its kind. The overall flow cytometric results revealed that with a fixed infection ratio, a higher proportion of BMDMs were infected from the susceptible line (7_2_) compared to the resistant line (6_1_). This might be an indication that macrophages play a significant role in exerting resistance to MDV in line 6_1_. Marek’s disease virus infection results in latency in resistant chicken lines in which the virus remains undetectable by the immune system [29]. In the present study, a low infection rate of BMDMs by MDV in the resistant line supports this hypothesis. Differential susceptibility or resistance between MHC-congenic chicken lines was also reported in the infection of macrophages with ILTV (infectious laryngotracheitis virus), another alpha-herpesvirus [43]. Macrophages are thought to inhibit MDV replication as they release NO (nitric oxide) through the increased activity of iNOS. An upregulation of iNOS pathway was revealed in this study in BMDMs of the resistant line during functional analysis of DE genes. This suggests a very rapid induction of the NO activity in BMDMs of the resistant line at 1 dpi. Nitric oxide is thought to be crucial for inhibiting MDV replication during the cytolylic and latent phases of infection in vivo, because an increased level of NO was observed in splenocyte cultures of MDV-infected resistant chickens [44]. Our study suggests that NO not only plays a role during the cytolytic and latent phases but may also act during the pre-cytolytic or entry phase of MDV infection.

The mRNA expression levels of two pro-inflammatory cytokines, *IL6* and *IL18*, in control and infected BMDMs from two inbred line, were measured in this study. In a previous in vivo MDV infection study [29], *IL6* and *IL18* were reported to be crucial factors in determining resistance or susceptibility to MD, as both cytokines were found to be upregulated in the splenocytes of susceptible birds (lines 7_2_ and P), but not in resistant chickens (lines 6_1_ and N). In partial agreement with the previous study, no significant expression of *IL6* was observed here in BMDMs of the resistant line, but this was the case in the susceptible line as well. An elevated expression of pro-inflammatory cytokines during the cytolytic phase is most likely related to increased pathology in susceptible chickens [29] which also correlates with another study where a higher expression of *IL18* was reported in caecal tonsils of susceptible chickens (line 7_2_) compared to resistant chickens (line 6_3_) [45]. In contrast, a significantly lower expression of *IL18* was measured in our study in BMDMs of the susceptible line following MDV infection. Macrophages, monocytes, and dendritic cells all express *IL18* [29] and one of the main roles of this cytokine is to enhance the activity of NK (natural killer) cells [46]. A lower NK cytotoxicity was detected previously in MD-susceptible compared to MD-resistant chickens [47,48]. In the present study, a reduced expression of *IL18* in MDV-infected BMDMs of the susceptible line might lead to decreased NK cell activity which in turn may play a role in formation of lymphomas in these birds, as a higher NK activity is more likely to be involved in antitumor responses [29].

An RNA-seq analysis of MDV-infected innate immune cells in vitro has not previously been carried out. To characterize the innate immune response to MDV infection, DE genes from control and infected BMDMs from both resistant and susceptible lines were analyzed and compared in this study. It was previously stated [16] that the resistance mechanism to MD in line 6_1_ birds could be due to the higher expression of key immune genes compared to that of line 7_2_ which in turn restricts disease progression in these chickens. In the present study, a higher level of immune gene expression was identified inherently in the BMDMs from the resistant line compared to that of the susceptible line, and differential gene expression was observed during macrophage responses between the two lines following MDV infection such as various interleukins, chemokines and their receptors being downregulated in the BMDMs from line 6_1_ while the opposite occurred in line 7_2_, suggesting the virus remains undetected by the immune system of resistant birds. This is in agreement with the statement that MDV persuades latency in resistant chickens where it is mostly not recognized by the host immune responses [29].

In our study, it was seen that genes involved in immune pathways such as TLR and JAK-STAT signaling were downregulated in BMDMs of line 6_1_ (resistant) during MDV infection. An important cellular pathway involved in the innate immune response is the JAK-STAT pathway [16] and it may have a role in the genetic basis of MD resistance [49,50]. A transcriptional upregulation of JAK-STAT and MAPK pathways was observed in MDV-infected CEFs of the same susceptible line but not in the resistant line, suggesting a role for these pathways in the expression of genes involved in cell survival and proliferation which might lead to MDV-induced transformation of cells in susceptible lines [51]. An activated JAK-STAT pathway is involved in cell survival and proliferation by nuclear translocation of an activated STAT dimer which results in transcription of genes involved in these processes [52]. In the present study, downregulation of the JAK-STAT pathway could therefore be a strategy of host cells (BMDMs of the resistant line) to induce cell death and subsequent restriction of virus transmission from cell-to-cell. Marek’s disease virus is strictly cell-associated, and no infectivity of virus is found outside the cell under in vitro conditions, suggesting cell-to-cell transmission of infectious virus particles [53]. Moreover, cellular projections between macrophages were observed by time-lapse confocal microscopy in our previous in vitro study [18], with these projections most likely being actin microfilaments [54]. Interestingly, TLR4-linked JAK2 signaling contributes to rearrangement of the cellular cytoskeleton (F-actin) during internalization of an intracellular microorganism, *Brucella abortus*, by BMDM in mice [55]. It can, therefore, be hypothesized that inactivation of such immune pathways in macrophages of MD-resistant chickens might lead to interruption of actin-mediated spread of MDV between cells which in turn results in limiting MDV infection in this line. However, further studies are needed to clarify this.

Downregulated genes expressed only in line 6_1_ infected BMDMs had significantly enriched *ZNF42* TF binding sites in their promoter region. This transcription factor, commonly known as myeloid zinc finger protein 1 (*MZF1*), is a zinc finger TF preferentially expressed in hematopoietic stem cells, myeloid progenitor cells, and in differentiated myeloid cells [56,57]. Several reports suggest *MZF1* as a putative tumorigenic protein [40,58]. To date, the role of *MZF1* TF binding sites in genes downregulated during MDV infection has not been evaluated. However, an enrichment of *HIC1* (hyper-methylated in cancer 1, a tumor-suppressor) binding sites was observed in the genes downregulated in MDV-infected splenocytes at 4 dpi in a previous study [16], and hence the authors suggested that MDV infection could block an anti-tumor mechanism long before the MDV oncogene (*Meq*) is expressed. In the present study, we see over-representation of TFBS for the putatively oncogenic MZF1 protein in genes downregulated after infection of resistant line 6_1_ BMDMs. This TF binding site was detected in genes downregulated during the host response in the MD-resistant line (6_1_) at 1 dpi, but not in the susceptible line (7_2_). This again appears to confirm that the resistant birds are able to suppress potential tumorigenic activity at an early stage post-infection. In addition, transcriptional repression by *MZF-1* requires FHL3 (four and a half LIM domain protein 3) as a cofactor [59] and FHL3 can also recruit a C-type binding protein (CtBP) as a co-repressor by which they regulate gene expression [60]. The interaction of CtBP with oncogene *Meq* plays a crucial role in MDV-induced lymphomas [61] and it was also speculated that by recruiting CtBP and its co-repressors, *Meq* might function in tumorigenesis and/or the establishment of latency in T cells [61]. MDV induces T cell latency in resistant lines, whereas it induces latency and lymphoma formation in susceptible birds. Although MDV transforms lymphocytes during its life cycle, the downregulation of macrophage genes with *MZF1* binding sites might have an influence on tumorigenesis when the infection transmits from macrophages to lymphocytes. Moreover, the *Meq* oncoprotein of the MDV vaccine strain (CVI988), which was used in this study, is unable to induce lymphoma as it has a 178 bp insertion which significantly diminishes its transactivation properties of this strain [62]. Therefore, further studies will be required to explore the exact role of *MZF1* in MD resistance.

Mapping DE genes located under known MDV QTL regions revealed putative candidate genes for MD resistance or susceptibility in both pre- and post-infection conditions. A gene involved in formation of tight junctions showed inherent upregulation in resistant line 6_1_ BMDMs (*CLDN5*), whereas *CD72*, which is involved in lymphoma of the small intestine, was highly expressed in MD-susceptible chickens. How tight junctions are regulated in macrophages of resistant chickens may be part of a mechanism by which cell-to-cell spread of virus is restricted in this line during infection [16]. A known tumor-suppressor gene, *RASEF* [63,64], was seen to be exclusively expressed in MDV-infected BMDMs of line 6_1_. The *HHLA2* gene, however, was highly expressed in BMDMs of MD-susceptible line 7_2_, which presumably implicates it in a novel immunosuppressive mechanism within the microenvironment of metastatic disease [65]. The ability of the resistant birds to suppress potential tumorigenic activity along with the expression of tumor-suppressor genes might be crucial in determining resistance to MD. However, no report of these genes has yet been published in relation to MD. Therefore, it will be interesting to explore their tentative role(s) in MD resistance or susceptibility in future studies.

## 5. Conclusions

Taken together, the data generated in this study support the hypothesis that resistance to MDV is most likely determined at the very early stage of infection during the innate immune response, with resistance mechanisms being deployed during early infection of macrophages. Many early defense methods are in play, such as the lessened ability of the virus to infect the resistant line compared to the susceptible line; differences in inherent gene expression between the two lines, resulting in differential host responses following infection, also promote resistance. Early anti-tumor mechanisms in the resistant line add to the strategies deployed by the host to fend off the effects of viral infection. We also highlight putative candidate genes for conferring resistance to MDV infection in macrophages.

## Figures and Tables

**Table 1:** Primers and probes for TaqMan qRT-PCR. F: forward primer; R: reverse primer.

RNA Target	Probe/Primer Sequence (5′-3′)
*28S*	Probe (FAM)-AGGACCGCTACGGACCTCCACCA-(TAMRA) F GGCGAAGCCAGAGGAAACT R GACGACCGATTTGCACGTC
*IL6*	Probe (FAM)-AGGAGAAATGCCTGACGAAGCTCTCCA-(TAMRA) F GCTCGCCGGCTTCGA R GGTAGGTCTGAAAGGCGAACAG
*IL18*	Probe (FAM)-CCGCGCCTTCAGCACGGATG-(TAMRA) F AGGTGAAATCTGGCAGTGGAAT R ACCTGGACGCTGAATGCAA

**Table 2:** The numbers of differentially expressed (DE) genes (*p* < 0.05; fold change > 2) used for functional analysis.

Category	Genes More Highly Expressed in Line 6_1_ Compared to Line 7_2_	Genes More Highly Expressed in line 7_2_ Compared to Line 6_1_
Host response in line 7_2_	**/**	1394↓ and 1133↑
Host response in line 6_1_	786↓ and 479↑	**/**
Inherent difference between lines	360	729
Difference between lines upon infection	401	555

## Supplementary Materials

The following are available online at http://www.mdpi.com/2073-4425/10/2/74/s1, Supplementary File 1: Table S1. Full lists of DEGs; Table S2. L6_HR_DAVID; Table S3. Viral read counts. Table S4. Differentially expressed viral genes; Table S5. L7_HR_DAVID; Table S6. MDV QTL candidates; Table S7. MDV QTL data; Table S8. Genes only expressed in L6; Table S9. Genes only expressed in L7. Supplementary File 2: Figure S1. MAC_inherent_pathways; Figure S2. MAC_inherent_biofunctions.

## Availability of RNA-seq Data and Materials

Data have been submitted to European Nucleotide Archive under accession number PRJEB29321.

## References

[1] Gimeno I.M. (2008). Marek’s disease vaccines: A solution for today but a worry for tomorrow?. Vaccine.

[2] Gavora J.S., Spencer J.L. (1979). Studies on genetic resistance of chickens to Marek’s disease—A review. Comp. Immunol. Microbiol. Infect. Dis..

[3] Von Krosigk C.M., McClary C.F., Vielitz E., Zander D.V. (1972). Selection for resistance to Marek’s disease and its expected effects on other important traits in White Leghorn strain crosses. Avian Dis..

[4] Rogers S.L., Kaufman J. (2008). High allelic polymorphism, moderate sequence diversity and diversifying selection for *B-NK* but not *B-lec*, the pair of lectin-like receptor genes in the chicken MHC. Immunogenetics.

[5] Lee S.H., Gitas J., Zafer A., Lepage P., Hudson T.J., Belouchi A., Vidal S.M. (2001). Haplotype mapping indicates two independent origins for the Cmv1s susceptibility allele to cytomegalovirus infection and refines its localization within the Ly49 cluster. Immunogenetics.

[6] Iizuka K., Naidenko O.V., Plougastel B.F., Fremont D.H., Yokoyama W.M. (2003). Genetically linked C-type lectin-related ligands for the NKRP1 family of natural killer cell receptors. Nat. Immunol..

[7] Vallejo R.L., Bacon L.D., Liu H.C., Witter R.L., Groenen M.A., Hillel J., Cheng H.H. (1998). Genetic mapping of quantitative trait loci affecting susceptibility to Marek’s disease virus induced tumours in F2 intercross chickens. Genetics.

[8] Yonash N., Bacon L.D., Witter R.L., Cheng H.H. (1999). High resolution mapping and identification of new quantitative trait loci (QTL) affecting susceptibility to Marek’s disease. Anim. Genet..

[9] McElroy J.P., Dekkers J.C., Fulton J.E., O’Sullivan N.P., Soller M., Lipkin E., Zhang W., Koehler K.J., Lamont S.J., Cheng H.H. (2005). Microsatellite markers associated with resistance to Marek’s disease in commercial layer chickens. Poult. Sci..

[10] Cole R.K. (1968). Studies on genetic resistance to Marek’s disease. Avian Dis..

[11] Lee L.F., Powell P.C., Rennie M., Ross L.J., Payne L.N. (1981). Nature of genetic resistance to Marek’s disease in chickens. J. Natl. Cancer Inst..

[12] Bumstead N., Sillibourne J., Rennie M., Ross N., Davison F. (1997). Quantification of Marek’s disease virus in chicken lymphocytes using the polymerase chain reaction with fluorescence detection. J. Virol. Methods.

[13] Haunshi S., Cheng H.H. (2014). Differential expression of Toll-like receptor pathway genes in chicken embryo fibroblasts from chickens resistant and susceptible to Marek’s disease. Poult. Sci..

[14] Liu H.C., Kung H.J., Fulton J.E., Morgan R.W., Cheng H.H. (2001). Growth hormone interacts with the Marek’s disease virus SORF2 protein and is associated with disease resistance in chicken. Proc. Natl. Acad. Sci. USA.

[15] Liu H.C., Niikura M., Fulton J.E., Cheng H.H. (2003). Identification of chicken lymphocyte antigen 6 complex, locus E (*LY6E*, alias *SCA2*) as a putative Marek’s disease resistance gene via a virus-host protein interaction screen. Cytogenet. Genome Res..

[16] Smith J., Sadeyen J.R., Paton I.R., Hocking P.M., Salmon N., Fife M., Nair V., Burt D.W., Kaiser P. (2011). Systems analysis of immune responses in Marek’s disease virus-infected chickens identifies a gene involved in susceptibility and highlights a possible novel pathogenicity mechanism. J. Virol..

[17] Calnek B.W. (2001). Pathogenesis of Marek’s disease virus infection. Curr. Top. Microbiol. Immunol..

[18] Chakraborty P., Vervelde L., Dalziel R.G., Wasson P.S., Nair V., Dutia B.M., Kaiser P. (2017). Marek’s disease virus infection of phagocytes: A *de novo* in vitro infection model. J. Gen. Virol..

[19] Jeurissen S.H., Scholten R., Hilgers L.A., Pol J.M., De Boer G.F. (1989). In situ detection by monoclonal antibody D-35.1 of cells infected with Marek’s disease virus that interact with splenic ellipsoid-associated reticulum cells. Avian Dis..

[20] Burgess S.C., Davison T.F. (2002). Identification of the neoplastically transformed cells in Marek’s disease herpesvirus-induced lymphomas: Recognition by the monoclonal antibody AV37. J. Virol..

[21] Bumstead N., Kaufman J., Davison F., Nair V. (2004). Genetic resistance to Marek’s disease. Marek’s Disease: An Evolving Problem.

[22] Cheng H.H., Perumbakkam S., Black Pyrkosz A., Subramaniam S., Preeyanon L., Dunn J., van Sambeek F., Ansah G., Muir W.M. The genetic architecture of genetic resistance to Marek’s Disease. Proceedings of the 8th European Symposium on Poultry Genetics.

[23] Wasson P. (2011). Development of Novel Virus Vectors for Influenza Vaccination. Ph.D. Thesis.

[24] Gimeno I., Silva R.F. (2008). Deletion of the Marek’s disease virus *UL41* gene (vhs) has no measurable effect on latency or pathogenesis. Virus Genes.

[25] Petherbridge L., Howes K., Baigent S.J., Sacco M.A., Evans S., Osterrieder N., Nair V. (2003). Replication-competent bacterial artificial chromosomes of Marek’s disease virus: Novel tools for generation of molecularly defined herpesvirus vaccines. J. Virol..

[26] Garceau V., Smith J., Paton I.R., Davey M., Fares M.A., Sester D.P., Burt D.W., Hume D.A. (2010). Pivotal Advance: Avian colony-stimulating factor 1 (CSF-1), interleukin-34 (IL-34), and CSF-1 receptor genes and gene products. J. Leukoc. Biol..

[27] Balic A., Garcia-Morales C., Vervelde L., Gilhooley H., Sherman A., Garceau V., Gutowska M.W., Burt D.W., Kaiser P., Hume D.A. (2014). Visualization of chicken macrophages using transgenic reporter genes: Insights into the development of the avian macrophage lineage. Development.

[28] Staines K., Hunt L.G., Young J.R., Butter C. (2014). Evolution of an expanded mannose receptor gene family. PLoS ONE.

[29] Kaiser P., Underwood G., Davison F. (2003). Differential cytokine responses following Marek’s disease virus infection of chickens differing in resistance to Marek’s disease. J. Virol..

[30] Wu Z., Rothwell L., Young J.R., Kaufman J., Butter C., Kaiser P. (2010). Generation and characterization of chicken bone marrow-derived dendritic cells. Immunology.

[31] Borowska D., Rothwell L., Bailey R.A., Watson K., Kaiser P. (2016). Identification of stable reference genes for quantitative PCR in cells derived from chicken lymphoid organs. Vet. Immunol. Immunopathol..

[32] FastQC: A Quality Control Tool for High Throughput Sequence Data. http://www.bioinformatics.babraham.ac.uk/projects/fastqc.

[33] Martin M. (2011). Cutadapt removes adapter sequences from high-throughput sequencing reads. EMBnet.journal.

[34] Li H., Handsaker B., Wysoker A., Fennell T., Ruan J., Homer N., Marth G., Abecasis G., Durbin R. (2009). 1000 Genome Project Data Processing Subgroup. The Sequence Alignment/Map format and SAMtools. Bioinformatics.

[35] Anders S., Pyl P.T., Huber W. (2015). HTSeq-a Python framework to work with high-throughput sequencing data. Bioinformatics.

[36] Robinson M.D., Smyth G.K. (2007). Moderated statistical tests for assessing differences in tag abundance. Bioinformatics.

[37] Robinson M.D., Smyth G.K. (2008). Small-sample estimation of negative binomial dispersion, with applications to SAGE data. Biostatistics.

[38] Huang da W., Sherman B.T., Lempicki R.A. (2009). Systematic and integrative analysis of large gene lists using DAVID bioinformatics resources. Nat. Protoc..

[39] Draghici S., Khatri P., Tarca A.L., Amin K., Done A., Voichita C., Georgescu C., Romero R. (2007). A systems biology approach for pathway level analysis. Genome Res..

[40] Mudduluru G., Vajkoczy P., Allgayer H. (2010). Myeloid zinc finger 1 induces migration, invasion, and in vivo metastasis through Axl gene expression in solid cancer. Mol. Cancer Res..

[41] Kodama H., Sugimoto C., Inage F., Mikami T. (1979). Anti-viral immunity against Marek’s disease virus infected chicken kidney cells. Avian Pathol..

[42] Powell P.C., Hartley K.J., Mustill B.M., Rennie M. (1983). Studies on the role of macrophages in Marek’s disease of the chicken. J. Reticuloendothel. Soc..

[43] Loudovaris T., Yoo B.H., Fahey K.J. (1991). Genetic resistance to infectious laryngotracheitis in inbred lines of White Leghorn chickens. Avian Pathol..

[44] Xing Z., Schat K.A. (2000). Inhibitory effects of nitric oxide and gamma interferon on in vitro and in vivo replication of Marek’s disease virus. J. Virol..

[45] Heidari M., Fitzgerald S.D., Zhang H. (2014). Marek’s disease virus-induced transient caecal tonsil atrophy. Avian Dis..

[46] Tsutsui H., Nakanishi K., Matsui K., Higashino K., Okamura H., Miyazawa Y., Kaneda K. (1996). IFN-gamma-inducing factor up-regulates Fas ligand-mediated cytotoxic activity of murine natural killer cell clones. J. Immunol..

[47] Garcia-Camacho L., Schat K.A., Brooks R., Bounous D.I. (2003). Early cell-mediated immune responses to Marek’s disease virus in two chicken lines with defined major histocompatibility complex antigens. Vet. Immunol. Immunopathol..

[48] Sharma J. (1981). Natural killer cells activity in chickens exposed to Marek’s disease virus: Inhibition of activity in susceptible chickens and enhancement of activity in resistant and vaccinated chickens. Avian Dis..

[49] MacEachern S., Muir W.M., Crosby S.D., Cheng H.H. (2012). Genome-wide identification and quantification of cis- and trans-regulated genes responding to Marek’s disease virus infection via analysis of allele specific expression. Front. Genet..

[50] Perumbakkam S., Muir W.M., Black-Pyrkosz A., Okimoto R., Cheng H.H. (2013). Comparison and contrast of genes and biological pathways responding to Marek’s disease virus infection using allele-specific expression and differential expression in broiler and layer chickens. BMC Genom..

[51] Subramaniam S., Preeyanon L., Cheng H.H. (2013). Transcriptional profiling of Meq-dependent genes in Marek’s disease resistant and susceptible inbred chicken lines. PLoS ONE.

[52] Boudny V., Kovarik J. (2002). JAK/STAT signalling pathways and cancer. Janus kinases/signal transducers and activators of transcription. Neoplasma.

[53] Denesvre C. (2013). Marek’s disease virus morphogenesis. Avian Dis..

[54] Richerioux N., Blondeau C., Wiedemann A., Rémy S., Vautherot J.F., Denesvre C. (2012). Rho-ROCK and Rac-PAK signalling pathways have opposing effects on the cell-to-cell spread of Marek’s disease virus. PLoS ONE.

[55] Lee J.J., Kim D.H., Kim D.G., Lee H.J., Min W., Rhee M.H., Cho J.Y., Watarai M., Kim S. (2013). Toll-like receptor 4-linked Janus kinase 2 signalling contributes to internalization of *Brucella abortus* by macrophages. Infect. Immun..

[56] Bavisotto L., Kaushansky K., Lin N., Hromas R. (1991). Antisense oligonucleotides from the stage-specific myeloid zinc finger gene MZF-1 inhibit granulopoiesis in vitro. J. Exp. Med..

[57] Morris J.F., Rauscher F.J., Davis B., Klemsz M., Xu D., Tenen D., Hromas R. (1995). The myeloid zinc finger gene, *MZF-1*, regulates the CD34 promoter in vitro. Blood.

[58] Hsieh Y.H., Wu T.T., Tsai J.H., Huang C.Y., Hsieh Y.S., Liu J.Y. (2006). PKC alpha expression regulated by *Elk-1* and *MZF-1* in human HCC cells. Biochem. Biophys. Res. Commun..

[59] Takahashi K., Matsumoto C., Ra C. (2005). FHL3 negatively regulates human high-affinity IgE receptor beta-chain gene expression by acting as a transcriptional co-repressor of *MZF-1*.. Biochem. J..

[60] Turner J., Nicholas H., Bishop D., Matthews J.M., Crossley M. (2003). The LIM protein FHL3 binds basic Krüppel-like factor/Krüppel-like factor 3 and its co-repressor C-terminal-binding protein 2. J. Biol. Chem..

[61] Brown A.C., Baigent S.J., Smith L.P., Chattoo J.P., Petherbridge L.J., Hawes P., Allday M.J., Nair V. (2006). Interaction of *Meq* protein and C-terminal-binding protein is critical for induction of lymphomas by Marek’s disease virus. Proc. Natl. Acad. Sci. USA.

[62] Ajithdoss D.K., Reddy S.M., Suchodolski P.F., Lee L.F., Kung H.J., Lupiani B. (2009). In vitro characterization of the Meq proteins of Marek’s disease virus vaccine strain CVI988. Virus Res..

[63] Maat W., Beiboer S.H., Jager M.J., Luyten G.P., Gruis N.A., van der Velden P.A. (2008). Epigenetic regulation identifies *RASEF* as a tumor-suppressor gene in uveal melanoma. Investig. Ophthalmol. Vis. Sci..

[64] Kaplon J., Hömig-Hölzel C., Gao L., Meissl K., Verdegaal E.M., van der Burg S.H., van Doorn R., Peeper D.S. (2014). Near-genomewide RNAi screening for regulators of BRAF(V600E)-induced senescence identifies *RASEF*, a gene epigenetically silenced in melanoma. Pigment Cell Melanoma Res..

[65] Koirala P., Roth M.E., Gill J., Chinai J.M., Ewart M.R., Piperdi S., Geller D.S., Hoang B.H., Fatakhova Y.V., Ghorpade M. (2016). *HHLA2*, a member of the B7 family, is expressed in human osteosarcoma and is associated with metastases and worse survival. Sci. Rep..

