# *Erratum:* Chakraborty, P. et al. Macrophages from Susceptible and Resistant Chicken Lines have Different Transcriptomes following Marek’s Disease Virus Infection. *Genes* 2019, *10*, 74

**DOI:** 10.3390/genes11070796

**Published:** 2020-07-15

**Authors:** Pankaj Chakraborty, Richard Kuo, Lonneke Vervelde, Bernadette M. Dutia, Pete Kaiser, Jacqueline Smith

**Affiliations:** 1 The Roslin Institute and R(D)SVS, University of Edinburgh, Easter Bush, Midlothian, EH25 9RG, UK; pcb23m@yahoo.com (P.C.); richard.kuo@roslin.ed.ac.uk (R.K.); lonneke.vervelde@roslin.ed.ac.uk (L.V.); bernadette.dutia@roslin.ed.ac.uk (B.M.D.); 2 Chittagong Veterinary and Animal Sciences University, Khulshi, Chittagong-4225, Bangladesh

The authors wish to make the following correction to their paper published in *Genes* [1]:

In the original article, the supplementary materials on page 14 were mistakenly listed as follows:

**Supplementary Materials**: The following are available online at http://www.mdpi.com/2073-4425/10/2/74/s1, Supplementary File 1: Table S1. Full lists of DEGs; Table S2. L6_HR_DAVID; Table S3. Viral read counts; Table S4. Differentially expressed viral genes; Table S5. L7_HR_DAVID; Table S6. MDV QTL candidates; Table S7. MDV QTL data; Table S8. Genes only expressed in L6; Table S9. Genes only expressed in L7. Supplementary File 2: Figure S1. MAC_inherent_pathways; Figure S2. MAC_inherent_biofunctions.

This should be replaced with:

**Supplementary Materials**: The following are available online at http://www.mdpi.com/2073-4425/10/2/74/s1, Supplementary File 1: Table S1. MDV QTL data; Table S2. Full lists of DEGs; Table S3. Viral read counts; Table S4. Differentially expressed viral genes; Table S5. L6_HR_DAVID; Table S6. L7_HR_DAVID; Table S7. Genes only expressed in L6; Table S8. Genes only expressed in L7; Table S9. MDV QTL candidates. Supplementary File 2: Figure S1. MAC_inherent_pathways; Figure S2. MAC_inherent_biofunctions.

The authors apologize for any inconvenience caused and the change does not affect the scientific results. The manuscript will be updated, and the original will remain online on the article webpage at https://www.mdpi.com/2073-4425/10/2/74.
